# Schistosomiasis - assessing progress towards the 2020 and 2025 global goals

**DOI:** 10.1056/NEJMoa1812165

**Published:** 2019-12-26

**Authors:** Arminder K. Deol, Fiona M. Fleming, Beatriz Calvo-Urbano, Martin Walker, Victor Bucumi, Issah Gnandou, Edridah M. Tukahebwa, Samuel Jemu, Upendo J. Mwingira, Abdulhakeem Alkohlani, Mahamadou Traore, Eugene Ruberanziza, Seydou Toure, Maria-Gloria Basanez, Michael D. French, Joanne P. Webster

**Affiliations:** 1 Schistosomiasis Control Initiative, Department of Infectious Disease Epidemiology, School of Public Health, Faculty of Medicine (St Mary's campus) Imperial College London, London, W2 1PG, UK; 2 Imperial College London, Department of Infectious Disease Epidemiology, School of Public Health, Faculty of Medicine (St Mary's campus) Imperial College London, London, W2 1PG, UK; 3 Royal Veterinary College, Department of Pathobiology and Population Sciences, University of London, Herts, AL9 7TA, UK; 4 London Centre for Neglected Tropical Disease Research, Department of Infectious Disease Epidemiology, School of Public Health, Faculty of Medicine (St Mary's campus), Imperial College London, London, W2 1PG, UK; 5 Ministry of Health Burundi, Avenue Pierre Ngendandumwe, Bujumbura, Burundi; 6 Ministry of Health Niger, Niamey, Niger; 7 Ministry of Health Uganda, Vector Control Division (MoH), Plot 15 Bombo Road, P.O Box 1661, Kampala, Uganda; 8 Ministry of Health Malawi, P. O. Box 30377, Lilongwe 3, Malawi; 9 Ministry of Health Tanzania, 6 Samora Machel Avenue, P.O.BOX 9083, 11478 Dar es salaam, Tanzania; 10 National Institute for Medical Research, Dar es Salaam; 11 Ministry of Health Yemen, P.O.Box 299, Sana'a, Republic of Yemen; 12 The Ministry of Public Health and Hygiene Mali, Bamako, Republic of Mali; 13 Neglected Tropical Diseases Unit, Malaria and Other Parasitic Diseases Division, Institute of HIV/AIDS, Disease Prevention and Control (IHDPC), Rwanda Biomedical Center (RBC), Ministry of Health, Kigali, Rwanda; 14 National Schistosomiasis Programme, Ministry of Health, Ouagadougou, Burkina Faso; 15 RTI International, 701 13th Street NW, Suite 750 Washington, DC, United States

## Abstract

**Background:**

With the vision of "a world free of schistosomiasis", the World Health Organization (WHO) has set ambitious goals, by 2020 and 2025, for, respectively, the control and elimination as a public health problem (EPHP) of this debilitating disease. As these milestones become imminent and if programmes are to succeed, it is crucial to gather quantitative evidence to support the existing universal approach of WHO programmatic guidelines.

**Methods:**

Multi-year cross-sectional data were collated and analysed from nine national schistosomiasis control programmes – eight in sub-Saharan Africa, and Yemen. Data were analysed by *Schistosoma* species *(Schistosoma mansoni, S. haematobium),* number of treatment rounds, overall prevalence and prevalence of heavy-intensity infection.

**Results:**

All but one country programme achieved control of morbidity targets for both schistosome species considerably sooner than current WHO guidelines project. Programmes with low baseline endemicity levels were more likely to reach control and EPHP targets. Intra-country variation was seen in the relationship between overall prevalence and that of heavy-intensity infection, and between treatment rounds, highlighting the challenges of using one metric to define control in all epidemiological settings.

**Conclusions:**

If countries follow the current guidelines, many programmes would need to continue beyond 2020. Our results suggest the need of a reduced timeframe from baseline to the next programmatic decision point (i.e. <5 years, rather than the proposed 5-10 years). This has important implications for national programmes, determining impact and resource allocations as well as indicating when to re-assess to determine the next treatment strategy.

## Introduction

Schistosomiasis is a parasitic neglected tropical disease (NTD), estimated to currently infect over 140 million people.^1,2^ The disease burden is greatest (at least 90%) in sub-Saharan Africa (SSA), where the main species causing human schistosomiasis are *Schistosoma mansoni* (intestinal schistosomiasis) and *S. haematobium* (urogenital schistosomiasis), transmitted through faeces and urine, respectively.^3,4^ Symptoms of schistosomiasis morbidity include anaemia, stunting, fever, genital lesions, and irreversible organ damage.^5–7^ Preventive chemotherapy (PC) with Praziquantel is the World Health Organization (WHO)-recommended strategy for the control of schistosomiasis and is primarily distributed to school-aged children (SAC) aged 5–15 years, who carry the highest infection burden and who can be reached efficiently through schools.^8^ The PC strategy is indicated by prevalence (estimated by initial parasitological assessment) at implementation unit level, usually district. Prevalence of infection less than 10% requires triennial PC, 10% to 49% biennial treatment, and 50% or greater annual treatment.^9^

The success of morbidity control in some countries ^10^ has led to a more ambitious vision of "a world free of schistosomiasis".^11^ The WHO has set goals for controlling schistosomiasis morbidity (defined as prevalence of heavy-intensity infection <5% aggregated *across* sentinel sites) by 2020 and achieving elimination as a public health problem (EPHP, defined as prevalence of heavy-intensity infection <1% *in all* sentinel sites) in all endemic countries by 2025. Complete interruption of transmission is a target in selected regions by 2025 (Figure 1).^11–13^ The WHO strategic plan provides guidance on how programmes can progress from control of schistosomiasis to EPHP and interruption of transmission.^11^ r

**Figure:**
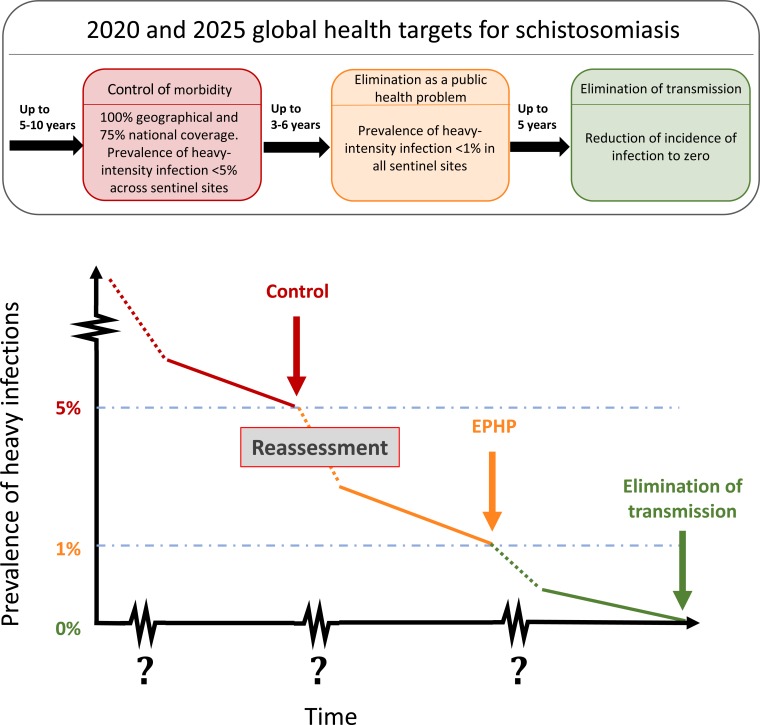


In practice, it is unlikely that the time lines for transitioning between goals will be uniform for all countries due to their epidemiological heterogeneity (Figure 1). Hence, there exists a need to analyse quantitative data, captured through programme monitoring, to validate and update these guidelines. Recent theoretical mathematical modelling work projects that the 2020 goal of morbidity control is likely obtainable for low and moderate prevalence settings, but will be missed in high-intensity settings with current treatment guidelines.^14^ We empirically addressed whether countries have already reached the 2020 and 2025 goals and if so, how many treatment rounds were required. Nationally representative cross-sectional epidemiological data for both *S. mansoni* and *S. haematobium* from nine countries were used. These data were made available by the national Ministries of Health of endemic countries. This study represents the first multi-country and multi-year empirical study to assess whether a one-size-fits-all approach is appropriate for guiding schistosomiasis treatment strategies to reach the WHO defined threshold criteria on morbidity control and EPHP.

## Materials and methods

### Data collation

Data were collated from the Schistosomiasis Control Initiative (SCI)-supported multi-year, cross-sectional treatment impact surveys in nine countries, which took place approximately six weeks prior to the following treatment round (i.e. just less than one year after the last treatment round for annual PC programmes and just less than two years after the last treatment round for biennial PC). . The inclusion criteria for were: i) countries where Ministries of Health were supported by the SCI; ii) having more than 2 years of impact survey data post baseline; and iii) cross-sectional data comprising SAC aged 5–15 years (Figure 2 and Table S1). Only epidemiological data available at SCI were analysed, so any further data points on the country programmes available from other sources were not included. Further details on the original surveys for this study can be found in the Supplementary Appendix.

**Figure:**
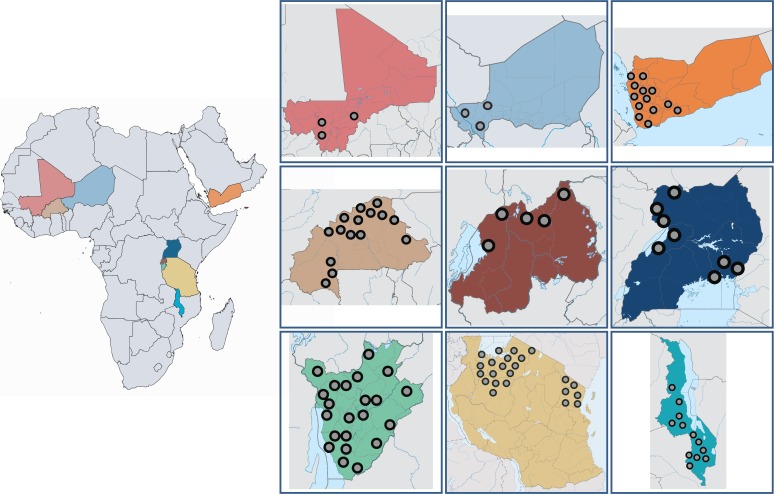


### Data analysis

The methods used to calculate sample sizes in each country programme were as currently employed at the SCI (Table S1). This provided the number of sentinel sites (schools) and children to be sampled within each site, powered to detect a pre-set difference in prevalence at a given administrative level for the country, accounting for clustering (a design effect) at the sentinel site level. Survey methods were standardised across countries. Standard Kato-Katz and urine filtration methods were used to detect *S. mansoni* and *S. haematobium* infection, respectively. The infection intensity category, i.e. the proportion of individuals with a given number of schistosome eggs per gram of faeces (epg) for *S. mansoni* (light intensity: 1-99 epg, moderate intensity: 100-399 epg and heavy intensity: ≥400 epg) or per 10 ml of urine for *S. haematobium* (light intensity: 1-50 eggs/10ml and heavy intensity: >50 eggs/10ml), and 95% confidence intervals (95% CIs), were calculated by treatment round, schistosome species, and country programme.^9^ Mean prevalence and 95% CIs were calculated to account for the clustering of the data at sentinel site level, using the R *survey* package.^15^ The point (mean) prevalence estimates were used for the comparison against WHO guidelines, since the guidelines do not suggest calculations of 95% CIs. We assessed whether the mean prevalence of heavy-intensity infection *across* sentinel sites fell to <5%, indicative of morbidity control, and/or <1% *in all* sentinel sites, indicative of EPHP.^11^ The overall prevalence and prevalence of moderate- plus heavy-intensity infection (S. *mansoni* only) was also estimated and compared with trends of heavy-intensity infection prevalence.

While the WHO guidelines only use prevalence of heavy-intensity infection as an indirect measure of morbidity (assuming morbidity is proportional to infection intensity), we included the combined measure of prevalence of moderate- plus heavy-intensity infection due to uncertainty in the appropriateness of egg count thresholds for intensity and because some degree of morbidity is likely to be caused by lighter infections.^16^

## Results

Baseline endemicity varied by species and country. *S. haematobium* ranged from 9.8% [95% CI: 6.0-15.5] prevalence in Malawi to 82.1% [95% CI: 70.1-90.0] in Mali-Segou.^9^ Prevalence for *S. mansoni* varied from 1.9% [95% CI: 0.5-6.9] in Malawi to 45.4% [95% CI: 35.6-55.7] in Uganda. Despite this heterogeneity, infection intensity in all countries fell following the first round of treatment to below, or within 0.8% of the 5% prevalence of heavy intensity threshold for control for *S. mansoni* infection and within 3.3% for *S. haematobium* (Figures 3 and 4 and Supplementary Appendix Figures S1 and S2).

**Figure:**
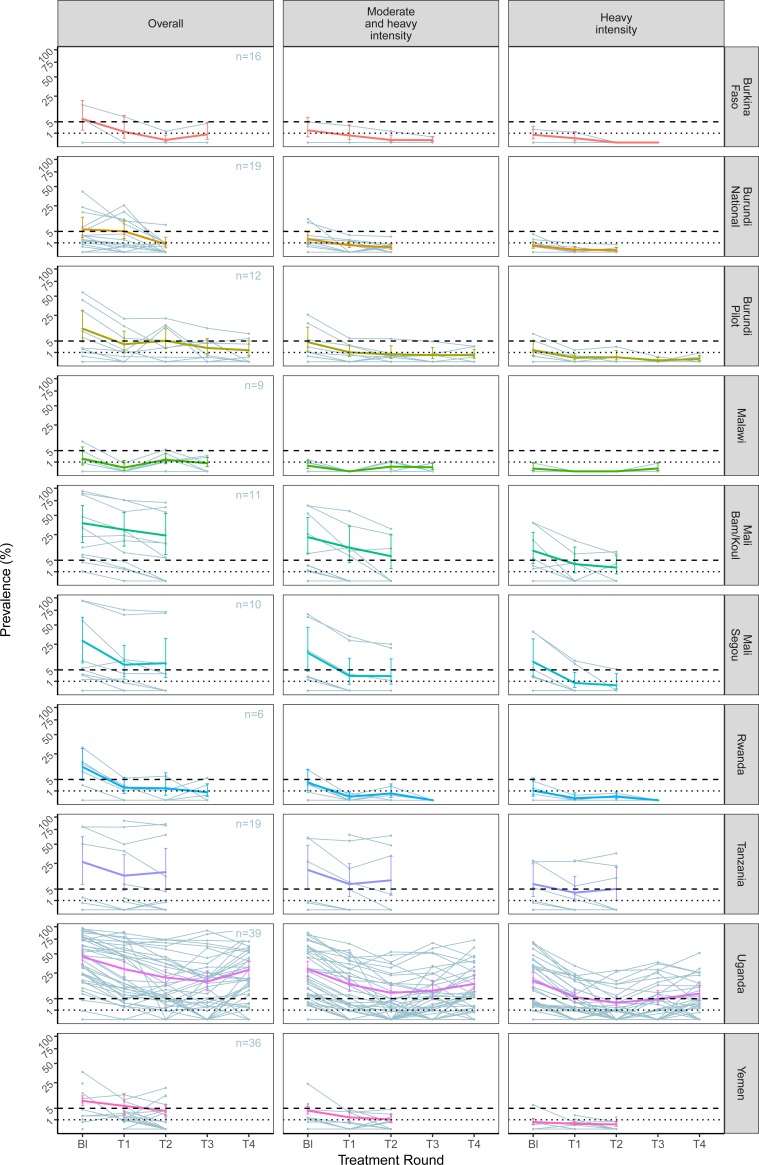


Treatment reduced the prevalence of heavy-intensity infection for both species to below 5% in all countries except Niger (5.4% [95% CI: 2.0-13.8]), which only marginally missed the metric for *S. haematobium* in the first treatment round (Figures 3 and 4 and Table 1). The more ambitious target of EPHP was only achieved for *S. mansoni* infection, and only in half of the country programmes. Moreover, Malawi had already reached EPHP for *S. mansoni* at baseline.

**Table 1:** Rounds of treatment required to reduce *Schistosoma mansoni* and *Schistosoma haematobium* infection to reach the World Health Organization's (WHO's) goal of morbidity control (<5% prevalence of heavy-intensity infection, aggregated across all sentinel sites) and elimination as a public health problem (EPHP, <1% prevalence of heavy-intensity infection in all sentinel sites). Baseline endemicity levels refer to the WHO prevalence category at country-level and the 95% CIs were calculated accounting for clustering of the data at the level of the sentinel sites.

Species	Baseline endemicity levels	Mean baseline prevalence % (95% CI)	Baseline prevalence of heavy-intensity infection % (95% CI)	Country	Frequency of treatment	Goal/s reached§	No. of treatment rounds (post-baseline)	*No. of treatment rounds for moderate- plus heavy-intensity prevalence*
*Schistosoma mansoni*	Low	6.5 (1.8-2.7)	0.7 (0.2-3.0)	Burkina Faso	Biennial	Control	0	*0*
						EPHP	2	*3*
	Low	6.0 (2.4-14.2)	0.5 (0.2-1.3)	Burundi National	Annual	Control	0	*0*
						EPHP	2	*Not yet reached*
	Low	12.7 (4.6-30.9)	1.5 (0.4-4.9)	Burundi Pilot	Annual	Control	0	*0*
						EPHP	3	*Not yet reached*
	Low	1.9 (0.5-6.9)	0.1 (0.0-0.9)	Malawi	Annual	Control	0	*0*
						EPHP	0	*1*
	Low	12.9 (4.6-31.2)	1.1 (0.2-5.7)	Rwanda	Annual	Control	0	*0*
						EPHP	1	*1*
	Low	9.2 (6.4-13.0)	0.6 (0.3-1.2)	Yemen	Biennial	Control	0	*0*
						EPHP	*Not yet reached*	*Not yet reached*
	Moderate	28.8 (8.9-62.6)	9.6 (2.5-27.5)	Mali-Segou	Annual	Control	1	*1*
						EPHP	*Not yet reached*	*Not yet reached*
	Moderate	38.8 (17.2-66.0)	10.6 (3.6-27.5)	Mali-Bamako/Koulikoro	Annual/Biennial	Control	1	*Not yet reached*
						EPHP	*Not yet reached*	*Not yet reached*
	Moderate	26.6 (7.4-62.1)	7.7 (2.1-24.7)	Tanzania	Annual	Control	1	*Not yet reached*
						EPHP	*Not yet reached*	*Not yet reached*
	Moderate	45.4 (35.6-55.7)	17.7 (11.7-25.8)	Uganda	Annual	Control	2	*Not yet reached*
						EPHP	*Not yet reached*	*Not yet reached*
*Schistosoma haematobium*	Low	9.8 (6.0-15.5)	2.2 (1.0-4.5)	Malawi	Annual	Control	0	*NA*
						EPHP	*Not yet reached*	*NA*
	Moderate	24.1 (14.1-38.0)	6.9 (3.2-14.4)	Tanzania	Annual	Control	1	*NA*
						EPHP	*Not yet reached*	*NA*
	Moderate	10.6 (6.5-16.8)	3.6 (2.1-6.3)	Yemen	Biennial	Control	0	*NA*
						EPHP	*Not yet reached*	*NA*
	High	56.2 (32.4-77.4)	25.2 (14.3-40.3)	Burkina Faso	Biennial	Control	1	*NA*
						EPHP	*Not yet reached*	*NA*
	High	70.0 (54.2-82.2)	20.8 (12.1-33.5)	Niger	Annual	Control	*Not yet reached*	*NA*
						EPHP	*Not yet reached*	*NA*
	High	82.1 (70.1-90.0)	44.0 (27.3-62.3)	Mali-Segou	Annual	Control	2	*NA*
						EPHP	*Not yet reached*	*NA*
	High	47.6 (33.5-62.1)	11.5 (6.8-18.6)	Mali-Bamako/Koulikoro	Annual/Biennial	Control	1	*NA*
						EPHP	*Not yet reached*	*NA*

**Figure:**
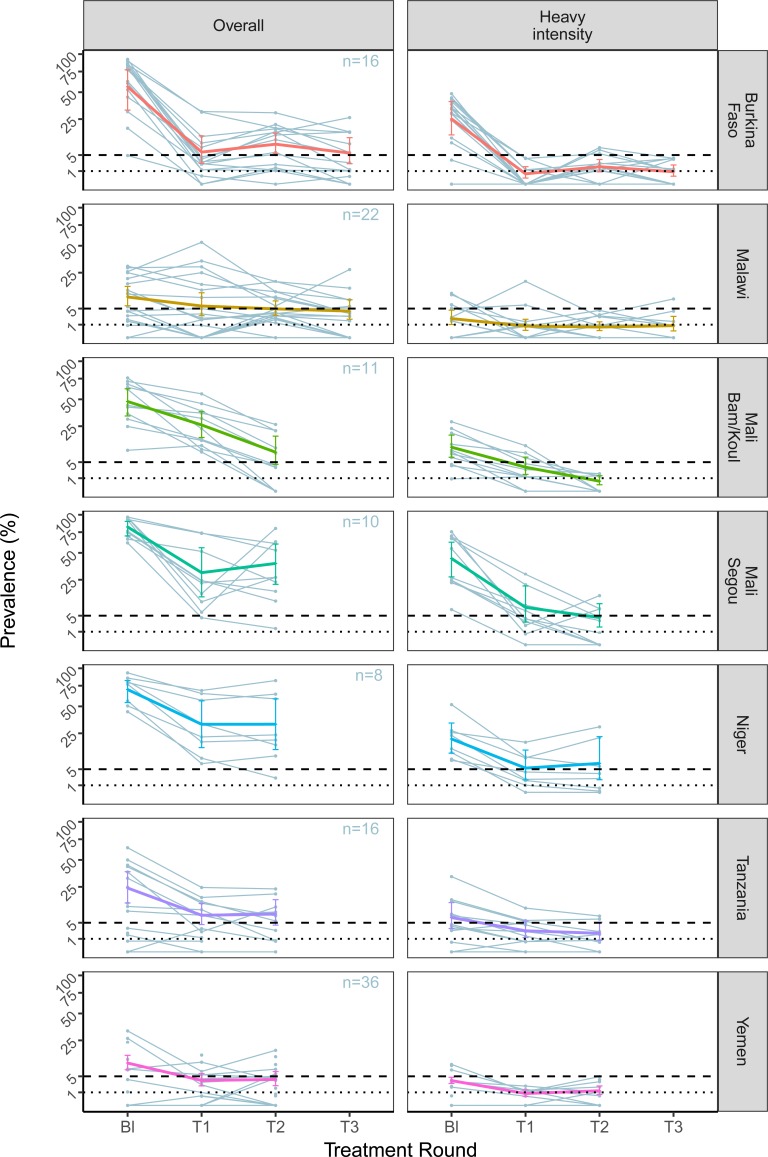


### Schistosoma mansoni

All ten country programmes reached the control of morbidity threshold after two rounds of treatment or fewer (Figure 3 and Table 1, Supplementary Appendix Figure S1). This included Uganda which had a relatively high baseline prevalence. However, a subsequent gradual increase in the prevalence of heavy-intensity infection to just over the 5% threshold was observed in Uganda after the third and fourth treatment rounds. Burkina Faso, Burundi (pilot and national programme) and Rwanda reached the EPHP threshold after three rounds or fewer (but note that these sites had a baseline mean prevalence of heavy-infection intensity already below the 5% morbidity control threshold).

When using the more conservative criterion of <1% and <5% prevalence of moderate- plus heavy-intensity infection to represent morbidity control (S. *mansoni* only), six country programmes were already below the <5% prevalence threshold at baseline and one further country programme (Mali-Segou) met this target after one round of treatment (Figure 3 and Table 1). Three country programmes achieved EPHP and only three out of ten country programmes failed to reach any target (control or EPHP) in the relatively short treatment period of the data currently available.

### Schistosoma haematobium

All countries had a baseline *S. haematobium* prevalence of heavy-intensity infection above 5%, except for Malawi and Yemen and, by the second treatment round, all except for Niger were below this threshold, meeting the control of morbidity criteria (Figure 4 and Table 1, Supplementary Appendix Figure S2). The prevalence of heavy-intensity infection in Niger fell following a single treatment round, from 20.8% [95% CI: 12.1-33.5] to 5.4% [95% CI: 2.0-13.8], only just missing the control of morbidity target.

Although three country programmes reached <1% heavy-intensity infection prevalence aggregated across sentinel sites (Figure 4), no countries reached this threshold in every sentinel site for *S. haematobium,* and thus did not meet the EPHP requirement.

## Discussion

The WHO provides guidance on the expected number of years of treatment to reach morbidity control and EPHP (5-10 years plus an additional 3-6 years, respectively). We demonstrate that these thresholds are often reached much sooner, whether with annual or biennial treatment. With the exception of *S. haematobium* in Niger, all programmes reached the morbidity control thresholds in two or fewer treatments rounds (between 1 and 2 years, depending on the frequency of PC). Notably, six country programmes started with a prevalence of heavy infection below 5% for *S. mansoni,* indicating that they were already at 'control' at baseline. The goal of EPHP for *S. mansoni* was reached by five programmes and required three or fewer treatment rounds (1 to 3 years). This prompts the question of what strategy to adopt in cases where the baseline prevalence of heavy-intensity infection already meets the control target. The *S. haematobium* areas had higher overall baseline infection levels and none reached the EPHP goals within the time horizon of this study. Endemically low prevalence countries, which also have lower baseline prevalence of heavy-intensity infection (≤1.5% for *S. mansoni),* achieved EPHP sooner than proposed by the guidelines. Through the analysis of extensive and nationally representative datasets from multiple countries, these results provide a crucial complement to recent theoretical modelling work which projects that achievement of control is possible in low and moderate prevalence settings and EPHP is possible in low prevalence areas.^14^ We recommend that this combination of programmatic data and mathematical models should be a blueprint for future activities as it utilizes the power of theoretical modelling to inform concretely programmatic goals and targets.

The case of Uganda illustrates that goals may be reached but are reversible (precise reasons for the Ugandan rebound are beyond the scope of the current study, but may reflect factors such as those relating to the influx of refugees, reduced compliance and/or changes in drug efficacy^17^). This is particularly relevant where programme stability is impeded, due to, for example, civil unrest in Burundi, war in Yemen. Figures 3 and 4 also highlight the variability between sentinel sites in each country, which need to be taken into consideration when looking at the country-level control of morbidity target proposed by the WHO. Additionally, the effectiveness of programme implementation may vary through time. It is, thus, important to define time periods over which control and elimination targets should be sustained to declare success and to be particularly vigilant to recrudescence of disease if elimination of transmission has not yet been achieved.

As expected, there was a strong positive association between overall infection prevalence and either the prevalence of heavy-intensity infection, or the prevalence of moderate- plus heavy-intensity infection (Supplementary Appendix Figure S3). There was substantial variation of data points between countries and treatment rounds which is likely caused by the heterogeneity of underlying adult parasite loads (perhaps due to variation in exposure among human hosts) such that the disease/morbidity prevalence varies substantially in settings with similar prevalence of infection. The magnitude of the change in infection following treatment varied substantially between country programmes. This emphasises the need for research on appropriate morbidity indicators. Once identified, they should be applied consistently across programmes and in guidelines.

It is not just heavy infections which lead to morbidity,^16^ therefore moderate plus heavy intensity infections were combined to form a more conservative metric of morbidity. When considering the aims of morbidity control and EPHP, since control thresholds may be reached relatively quickly, it would be worth considering this metric in the meantime, to include a larger population group potentially suffering from morbidity.

An important limitation of this study is the absence of information on specific treatment coverage which can, in general, vary substantially among national scale PC programmes (see Supplementary Appendix). Other information such as migration patterns, school-enrolment and attendance rates may also influence the effectiveness of a PC programme and explain variation among study areas. Detailed information on these factors has not been routinely collected by the SCI. This is common to NTD programmes and we recommend that, in future, the scope of data collection should be enhanced and incorporated into routine data collection protocols. This additional information would support the interpretation of epidemiological data when evaluating the impact and effectiveness of PC programmes.

More than half of the programmes had sentinel sites of mixed *S. mansoni* and *S. haematobium* infections. In this study, the species were analysed independently; however, some areas may have had higher infection prevalence when both are combined, or underlying interactions occurring.^18^ These issues require further clarification in the guidelines. China (endemic species *S. japonicum)* and Brazil (S. *mansoni)* have shown great progress towards achieving interruption of transmission, particularly considering the added challenge of multiple animal reservoirs of *S. japonicum.* This highlights that further integration of other practices such as clean water and sanitation, treatment of the animal population and snail control are required, all of which are still lagging in the much of the SSA settings.

It is necessary to mention briefly some of the key factors which highlight the need for the guidelines to be updated. The metrics and definitions for control and elimination of schistosomiasis-related morbidity use the egg-count intensity cut-offs. However, these metrics, definitions and egg-count cut-offs are an imperfect measure of infection and the relationship between morbidity and egg counts needs to be carefully and urgently addressed. The adult and pre-SAC population are generally not actively monitored and data on SAC are currently used as a proxy for the situation in the wider community. However, it is unrealistic to declare EPHP (and in some cases even control) with this unmonitored reservoir in the population. Suitability of the currently recommended diagnostic tools, upon which the guidelines are based, also need to be promptly assessed. Studies are actively looking at the feasibility and cost-effectiveness of alternative and more accurate diagnostics for large-scale use, particularly as diagnostic sensitivity decreases with reduced infection intensity.^19–24^ Another critical area is the hotspot phenomenon – a blanket catchall term used to describe areas of persistent infection despite multiple rounds of treatment. Guidance on defining and managing hotspot areas, especially in countries which are otherwise on target for the 2020/2025 goals, are presently lacking, but studies are aiming to address this.^25–27^ Additional analysis of available data, the ability to collect such information routinely as part of large-scale control programmes, and further research are required to establish a robust evidence base for these (or updated) targets, which will be critical especially as countries move towards interruption of transmission. What has not yet been addressed is that there will still be true morbidity in the community even after the targets have been reached, since schistosomiasis morbidity can continue many years after infection has ceased (e.g. genital schistosomiasis, hepatosplenomegaly, etc.). Achieving true morbidity control and elimination would thus require the redefining of morbidity control (not to be completely dependent on egg-output) and incorporation of new strategies that address long-term morbidity, such as the SAFE (Surgery, Antibiotics, Facial Cleanliness and Environmental improvement) adopted as the recommended strategy for trachoma elimination.^28^

Our study analyses the most extensive datasets available to assess the timeframes in the WHO's guidelines for control and elimination of schistosomiasis. In conclusion, if the indicative timelines to transition to the next control or elimination goal are accurate, these will take many countries beyond the 2020 and 2025 targets. Where countries have <5% heavy-intensity prevalence at baseline, it is unclear whether they should aim immediately for EPHP or continue to treat as per guidelines for 5-10 years. The study's key messages are that countries often achieve morbidity control following very few treatment rounds, and that the universal timeline currently recommended is not appropriate for all programmes, and will be affected by baseline endemicity, schistosome species, and context-specific relationship between infection and morbidity. Outputs from analysis of empirical and modelling data can be used to update these timelines. This will allow more useful programmatic decision-making tools and more accurate projections of progress against schistosomiasis at the national, regional, and global level as we move towards the 2020 and 2025 goals.
