# Supplementary material for: Schistosomiasis - assessing progress towards the 2020 and 2025 global goals
Source: N Engl J Med. Author manuscript; Available in PMC 2019 Dec 26. (PMC6785807; doi:10.1056/NEJMoa1812165)
Supplement: Supplementary file 1 [file NEJM-2019-1812165-s1.pdf]

Supplementary Appendix

Table of Contents

The national control programmes ***2***

Sentinel site selection process of the original SCI programmes in brief **2**

Field staff training **2**

Data collection **2**

Treatment programme **3**

Overall country-level trends towards control of schistosomiasis morbidity: Schistosoma mansoni ***4***

Overall country-level trends towards control of schistosomiasis morbidity: Schistosoma haematobium ***5***

Relationship between prevalence of heavy-intensity and overall prevalence ***6***

Data used in this study ***7***

References ***7***

The national control programmes

Sentinel site selection process of the original SCI programmes in brief

As part of the Monitoring and Evaluation (M&E) component of the schistosomiasis control programmes, the sentinel sites for impact monitoring were selected to be fully representative of the endemicity levels of the wider regions/countries under study. Full details have been published elsewhere and see Table S1. In summary, initial mapping exercises were performed by suitably trained technicians per country prior to baseline treatment. Results from mapping evaluated endemicity levels and informed sentinel site selection for the programme M&E. These sentinel sites were chosen at random across areas of different endemicity levels to represent national level changes. Study schools within these sites were chosen using a random-number generator computer algorithm. The level of precision of 10% (whereby the sample size was sufficient to identify any change in overall prevalence of at least 10% of the baseline figure) was selected.

The number of sentinel sites per country was inherently different, reflecting the differing baseline endemicities. As standard, the same sentinel sites were used each year in the impact surveys to provide a direct comparison of treatment impact after each round.

Extensive effort was made in selecting, training and M&E coverage to treat communities with sentinel sites in exactly the same way as standard communities in order prevent any bias. Drug distributors were not part of the sentinel site surveys.

Field staff training

Training for the sentinel sites was conducted by Ministry of Health staff in each country with the support of the Schistosomiasis Control Initiative (SCI). Training topics included parasitological diagnostic methods (Kato Katz, urine filtration, urine dipsticks), participant selection, fieldwork logistics, and data entry. Training materials used a standardised training framework to maintain consistency and quality. The personnel trained included representatives of the central Ministry of Health and district health staff in each country.

The standard parasitological methods for detecting intestinal schistosomiasis (Kato Katz) and urogenital schistosomiasis (urine filtration) were conducted throughout the portfolio of countries.[1,2] These field-appropriate tests were performed by fully-trained personnel at the field sites. Senior technicians verified slide readings from a randomly-selected sample of 10% of the samples, as detailed above, to maximise consistency in slide reading accuracy.

Data collection

Monitoring and Evaluation data collection was undertaken at (pre-treatment) baseline (prior to the start of national control programme activities) and subsequently immediately prior to (up to 6 weeks) each treatment round in the following years. In order to maintain consistency between the sentinel sites and the national programme, individuals in the sentinel sites were offered treatment at the time of the national control programme activities. It is worth noting that the programme managers for three countries (Burkina Faso, Niger and Uganda) state that some survey teams may have offered treatment to infected participants prior to the Mass Drug Administration (MDA), although this is expected to have minimal impact on the overall population infection level due to the relatively large sample sizes and the close succession of treatment for these individuals.

Within each country, the objectives of the survey were first explained to the local village chiefs and political and religious authorities, alongside teachers and parents, who gave their written or verbal consent based on the institutional review board (IRB) or MoH programme practices within the country, to participate in the study. Verbal and, wherever possible also written, consent were also given by the individual school children before recruitment into the survey.

Treatment programme

Treatment was funded through various agencies (see "Acknowledgments" section of the main manuscript), led by the national Ministries of Health and conducted through school platform through teachers and/or community drug distributors.

Treatment coverage was determined through routine national treatment coverage reports and independent coverage evaluation surveys from across the countries at the implementation unit (IU) level. Data from these sources demonstrated that despite intra and inter-variation in treatment coverage at the IU level, coverage in school-age children, on average, achieved the WHO target of a minimum of 75% or above.

Overall country-level trends towards control of schistosomiasis morbidity: *Schistosoma mansoni*

Figure S1. Temporal changes in mean prevalence of *Schistosoma mansoni* infection in school-aged children (SAC) aggregated across sentinel sites from each country. The black dashed lines show the 5% cut-offs for prevalence of heavy-intensity infection indicating control of morbidity. Rows represent the prevalence of overall, moderate- plus heavy-intensity and heavy-intensity infection, showing the mean prevalence at country-level, and the error bars show the 95% confidence intervals, accounting for clustering of the data at the level of the sentinel sites.

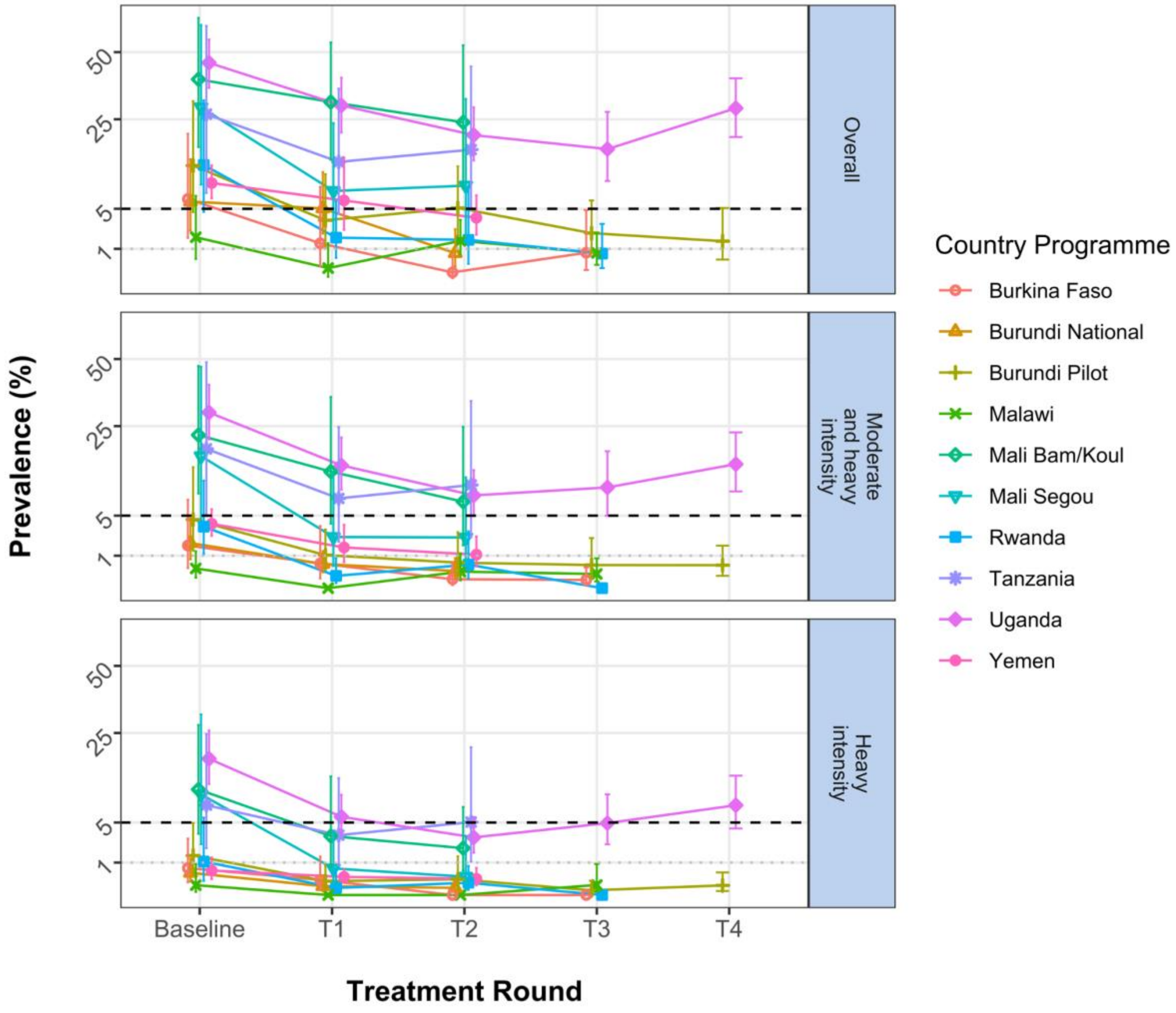

Overall country-level trends towards control of schistosomiasis morbidity: *Schistosoma haematobium*

Figure S2. Temporal changes in prevalence of *Schistosoma haematobium* infection in school-aged children (SAC) aggregated across sentinel sites from each country. The black dashed lines show the 5% cut-offs for prevalence of heavy-intensity infection indicating control of morbidity. Rows represent the prevalence of overall and heavy-intensity infection, showing the mean prevalence at country-level, and the error bars show the 95% confidence intervals, accounting for clustering of the data at the level of the sentinel sites.

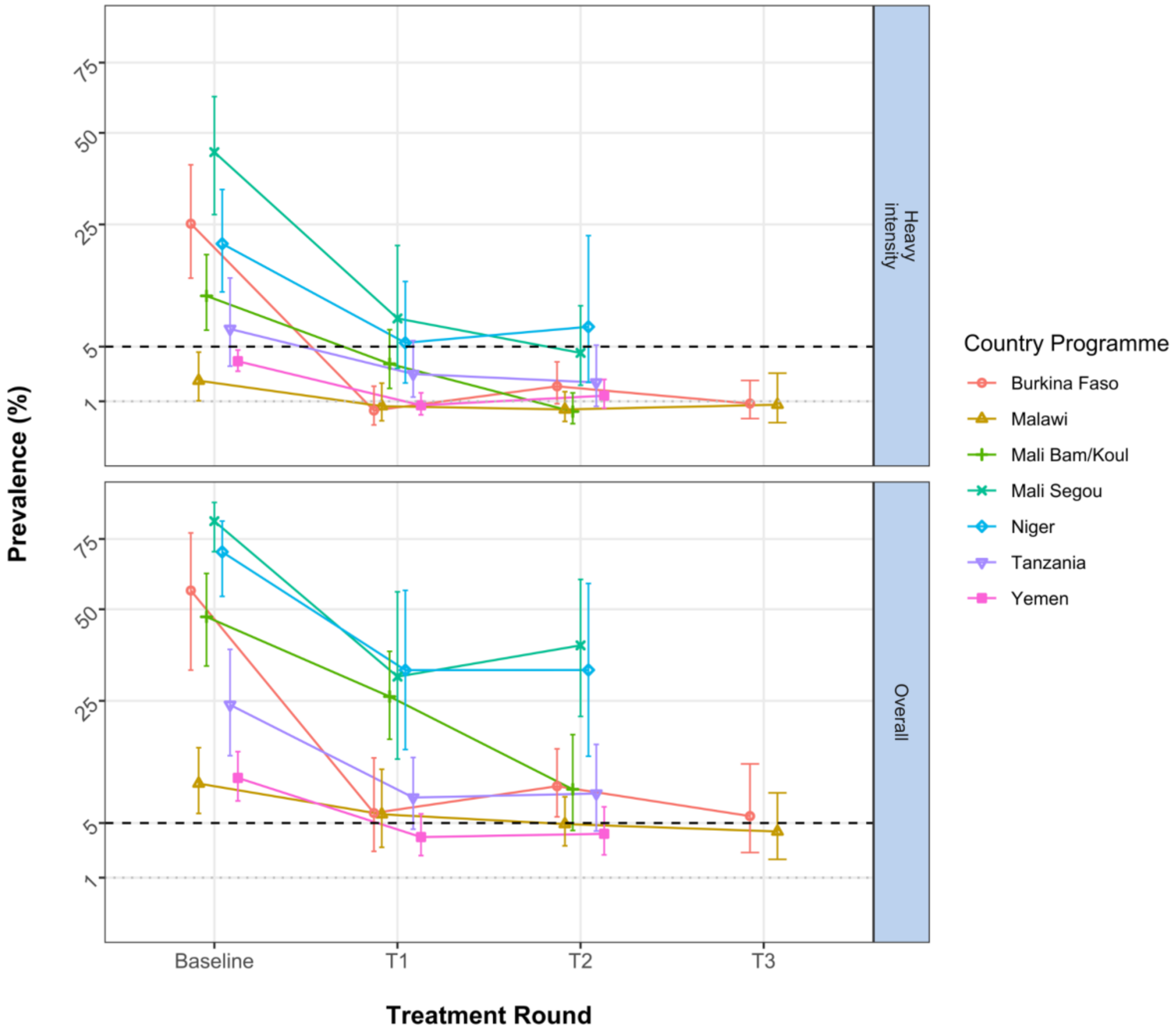

Relationship between prevalence of heavy-intensity and overall prevalence

Figure S3. Relationship between prevalence of heavy intensity infection (and moderate and heavy intensity infection for *Schistosoma mansoni*) and overall prevalence in school-aged children (SAC) across all sentinel sites by species. Best fit (black line) and standard errors (grey band) were obtained by fitting a loess smoothed line. Colours represent treatment round and shapes represent country programme.

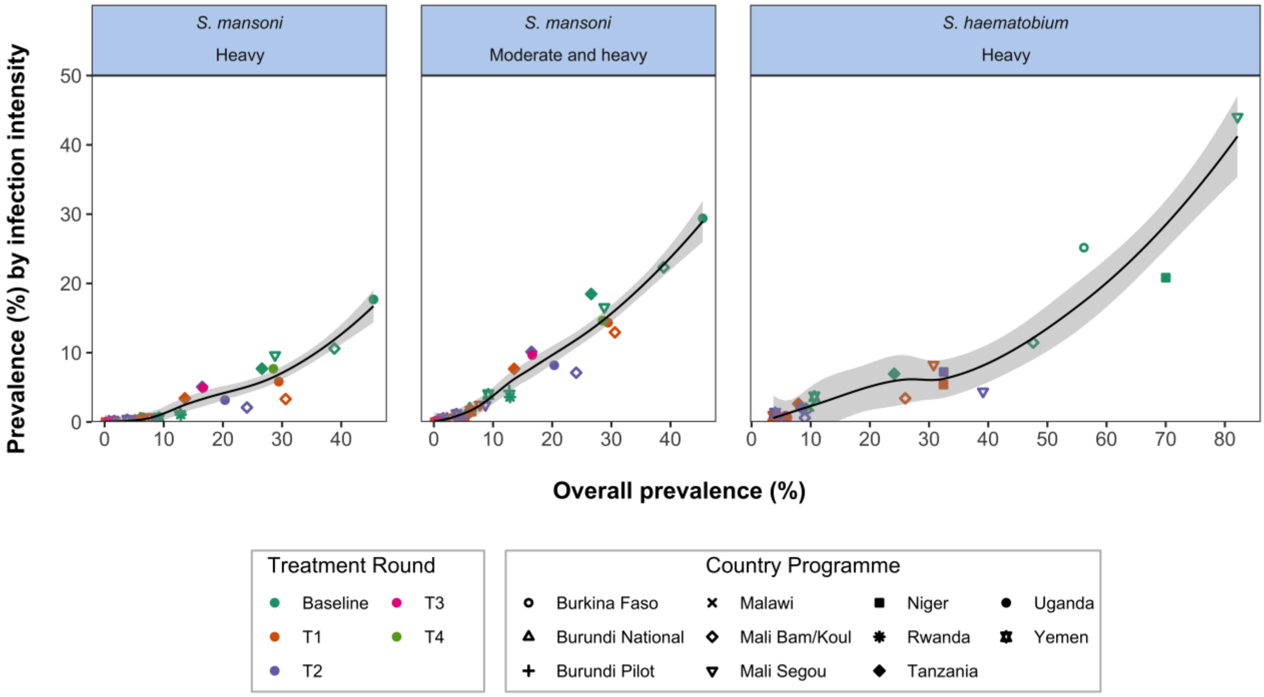

For both schistosome species, the relationship between prevalence of heavy-intensity (and moderate- plus heavy-intensity) infection *vs* overall infection prevalence was non-linear (Figure S3). A wide range of prevalence values within and between treatment rounds corresponded to <5% heavy-intensity infection (and moderate- plus heavy-intensity) infection.

Data used in this study

Table S1. Data used in this study from SCI-assisted programmes

Country programme	Start year	Endemic parasite species	Baseline endemicity (*spp.*)	Treatment frequency	Number of sentinel sites	Average number of individuals per sentinel site (SD)	Ref.
**Burkina Faso**	2004	*S. m.*<br>*S. h.*	Low (*S. m.*)<br>High (*S. h.*)	Biennial	16	94 (48)	3
**Burundi National***	2008	*S. m.*	Low (*S. m.*)	Annual	19	307 (78)	4
**Burundi Pilot***	2007	*S. m.*	Low (*S. m.*)	Annual	12	319 (65)	4
**Malawi**	2012	*S. m.*<br>*S. h.*	Low (*S. m.*)<br>Low (*S. h.*)	Annual	22	110 (15)	*(Pers. Comm.)*
**Mali-Segou***	2004	*S. m.*<br>*S. h.*	Moderate (*S. m.*)<br>High (*S. h.*)	Annual	10	103 (26)	5,6
**Mali-Bamako/ Koulikoro***	2004	*S. m.*<br>*S. h.*	Moderate (*S. m.*)<br>High (*S. h.*)	Mixed	11	76 (24)	5,6
**Niger**	2004	*S. h.*	High (*S. h.*)	Annual	8	285 (83)	7
**Rwanda**	2008	*S. m.*	Low (*S. m.*)	Annual	6	254 (103)	*(Pers. Comm.)*
**Tanzania**	2005	*S. m.*<br>*S. h.*	Moderate (*S. m.*)<br>Moderate (*S. h.*)	Annual	21	124 (62)	7
**Uganda**	2003	*S. m.*	Moderate (*S. m.*)	Annual	39	80 (45)	8
**Yemen**	2010	*S. m.*<br>*S. h.*	Low (*S. m.*)<br>Moderate (*S. h.*)	Biennial	36	508 (941)	*(Pers. Comm.)*

S. m. = *Schistosoma mansoni*; *S. h.* = *Schistosoma haematobium*; Pers. Comm. = personal communication. *Burundi and Mali were split into two segments in this study: 'Burundi Pilot' (containing data from twelve sentinel sites) and 'Burundi National' (data from nineteen sentinel sites), where the surveys began in different years, and 'Mali-Segou' (one sentinel region, ten sentinel site schools) and 'Mali-Bamako/Koulikoro' (two regions, eleven sentinel site schools) where different treatment strategies were implemented. SD = standard deviation.

References

1. WHO | Urine Filtration. [cited 2019 Jun 3]. Available from: http://www.ntdsupport.org/resources/immunochromatographic-card-test-ict-

2. WHO | Basic laboratory methods in human parasitology (Examination of fecal and urine specimens) [Internet]. World Health Organization; 2017 [cited 2019 Jun 3]. Available from: https://www.who.int/neglected_diseases/preventive_chemotherapy/Basic_Lab_methods_in_human_parasitology/en/index2.html

3. Koukounari A, Gabrielli AF, Touré S, et al. Schistosoma haematobium Infection and Morbidity Before and After Large-Scale Administration of Praziquantel in Burkina Faso. J Infect Dis 2007;196(5):659–69.

4. Ortu G, Assoum M, Wittmann U, et al. The impact of an 8-year mass drug administration programme on prevalence, intensity and co-infections of soil-transmitted helminthiases in Burundi. Parasites and Vectors 2016;9(1):1–17.

5. Koukounari A, Donnelly CA, Sacko M, et al. The impact of single versus mixed schistosome species infections on liver, spleen and bladder morbidity within Malian children pre- and post-praziquantel treatment. BMC Infect Dis 2010;10(1):227.

6. Koukounari A, Sacko M, Keita AD, et al. Assessment of ultrasound morbidity indicators of schistosomiasis in the context of large-scale programs illustrated with experiences from Malian children. Am J Trop Med Hyg 2006;75(6):1042–52.

7. Fenwick A, Webster JP, Bosque-Oliva E, et al. The Schistosomiasis Control Initiative (SCI): rationale, development and implementation from 2002-2008. Parasitology 2009;136(13):1719–30.

8. French MD, Churcher TS, Gambhir M, et al. Observed reductions in Schistosoma mansoni transmission from large-scale administration of praziquantel in Uganda: a mathematical modelling study. PLoS Negl Trop Dis 2010;4(11):e897.
